# Making a Cold Tumor Hot: The Role of Vaccines in the Treatment of Glioblastoma

**DOI:** 10.3389/fonc.2021.672508

**Published:** 2021-05-10

**Authors:** Stephen C. Frederico, John C. Hancock, Emily E. S. Brettschneider, Nivedita M. Ratnam, Mark R. Gilbert, Masaki Terabe

**Affiliations:** ^1^ Neuro-Oncology Branch, CCR, NCI, National Institutes of Health, Bethesda, MD, United States; ^2^ Ludwig Institute for Cancer Research, University of Oxford, Oxford, United Kingdom

**Keywords:** glioblastoma, vaccine, dendritic cells, peptide, tumor antigen, neoantigen, T cells, heat shock protein

## Abstract

The use of immunotherapies for the treatment of brain tumors is a topic that has garnered considerable excitement in recent years. Discoveries such as the presence of a glymphatic system and immune surveillance in the central nervous system (CNS) have shattered the theory of immune privilege and opened up the possibility of treating CNS malignancies with immunotherapies. However, despite many immunotherapy clinical trials aimed at treating glioblastoma (GBM), very few have demonstrated a significant survival benefit. Several factors for this have been identified, one of which is that GBMs are immunologically “cold,” implying that the cancer does not induce a strong T cell response. It is postulated that this is why clinical trials using an immune checkpoint inhibitor alone have not demonstrated efficacy. While it is well established that anti-cancer T cell responses can be facilitated by the presentation of tumor-specific antigens to the immune system, treatment-related death of GBM cells and subsequent release of molecules have not been shown to be sufficient to evoke an anti-tumor immune response effective enough to have a significant impact. To overcome this limitation, vaccines can be used to introduce exogenous antigens at higher concentrations to the immune system to induce strong tumor antigen-specific T cell responses. In this review, we will describe vaccination strategies that are under investigation to treat GBM; categorizing them based on their target antigens, form of antigens, vehicles used, and pairing with specific adjuvants. We will review the concept of vaccine therapy in combination with immune checkpoint inhibitors, as it is hypothesized that this approach may be more effective in overcoming the immunosuppressive milieu of GBM. Clinical trial design and the need for incorporating robust immune monitoring into future studies will also be discussed here. We believe that the integration of evolving technologies of vaccine development, delivery, and immune monitoring will further enhance the role of these therapies and will likely remain an important area of investigation for future treatment strategies for GBM patients.

## Introduction

Glioblastoma (GBM) is one of the most lethal primary brain malignancies, with a median overall survival of 14-17 months despite intervention with both surgery and chemo-radiation therapy (1, 2). In recent years, there has been hope that immunotherapy would be a promising new approach to treat this devastating disease. Since 2011, a new wave of immune checkpoint inhibitors (ICIs) such as anti-CTLA-4 and anti-PD-1/PD-L1 monotherapies have been approved for melanoma, non-small cell lung cancer, and other solid malignancies outside of the CNS (3). The hypothesis of immune privilege in the CNS has begun to weaken, making immunotherapy a possibility for the treatment of GBM and other CNS cancers (4–6). Early murine studies conducted to test the efficacy of anti-PD-1, anti-PD-L1, and anti-CTLA-4 using orthotopic, syngeneic GBM models were very promising, demonstrating long-term tumor eradication using single-agent therapy and a cure rate of 75% when combining anti-CTLA-4 with anti-PD-1 (7). These results led to CheckMate 143: the first major clinical trial for immunotherapy in GBM (1). This phase III trial tested the survival benefit of anti-PD-1 monotherapy in 369 patients with recurrent GBM (8). Unfortunately, the outcome of the trial was disappointing as no significant difference was found between patients receiving treatment with anti-PD-1 in comparison to those receiving the standard of care (8). To date, immune checkpoint monotherapy has not been proven to be successful in the treatment of GBM clinically (1). Also, no phase III clinical trial with any immunotherapy approach has demonstrated benefit in GBM patients (9). One of the primary reasons for this failure is the ability of GBM tumor cells to induce immune suppression (1, 9, 10), which is why combination of different therapies may yield better results (1, 9). A call to action has now been made to develop new therapies that can provide patients with improved OS.

The mechanisms of GBM immunosuppression are multifaceted, with effects propagated both locally and systemically. At the local level, tumors can recruit regulatory T cells and induce tumor-associated macrophages to cause T cell apoptosis (4). Immunosuppressive cytokines such as IL-10, TGF-beta, and CCL2 are also secreted (11). GBM uses metabolites such as kynurenine to polarize macrophages to an anti-inflammatory phenotype (1). These mechanisms result in the majority of immune cell infiltrates being composed of immunosuppressive MDSCs and tumor-associated macrophages (11). On a systemic level, intracranial tumors can cause sequestration of T cells in the bone marrow (12). It is speculated that this is induced through the loss of the sphingosine-1-phosphate receptor 1 (S1P1) from the T cell surface, a G-protein-coupled receptor that plays a vital role in lymphocyte trafficking (12). Furthermore, recent evidence has identified meningeal lymphatic drainage that lies between the brain parenchyma and cervical lymph nodes: the “glymphatic system” (13, 14). Meningeal lymphatics play a role in the control of immune surveillance of the CNS (15). It is possible that GBM disrupts this drainage and thus hinders antigen flow and immune cell trafficking (13, 15, 16). GBMs are thus known as “cold” tumors, which have few or no lymphocyte infiltrates (11). One promising strategy to “heat up” a cold tumor is to promote a robust anti-tumor T cell response through the use of vaccines.

In 1953, observations in radiation oncology highlighted a phenomenon that has become known as the abscopal effect (17, 18). The idea refers to the systemic regression of tumors and metastases in non-radiated areas outside of the primary localized radiation field (17, 18). It is hypothesized that radiation induces the release of tumor antigens which then prime the immune system for an anti-tumor response (17). This observation inspires the possibility of stimulating the immune system using exogenously introduced antigens and is the basis for the generation and use of anti-cancer vaccines. Ideally, systemic induction of an anti-cancer T cell response by vaccines can lead to increased trafficking to the tumor site. One could theoretically “heat up” an immunologically “cold” tumor. Thus in the case of cancer, vaccinations are therapeutic rather than prophylactic (9).

It has become clear that GBMs are complex and heterogeneous, evolving before, during, and after treatment (4). Given the vast inter- and intra-tumoral heterogeneity and multiple facets of immunosuppression provided by GBM, a single target approach may not be effective. The pooled mechanisms of multiple distinct therapies will be required. One important observation in GBM is that increased levels of inflammation in and around the tumor site induces increased PD-L1 expression (10). Thus, it is anticipated that the combination of vaccines and PD-1/PD-L1 targeted therapy would be synergistic in overcoming GBM immunosuppression. Combinations of ICIs with other therapies is increasingly being tested in clinical trials (1).

**Figure 1:**
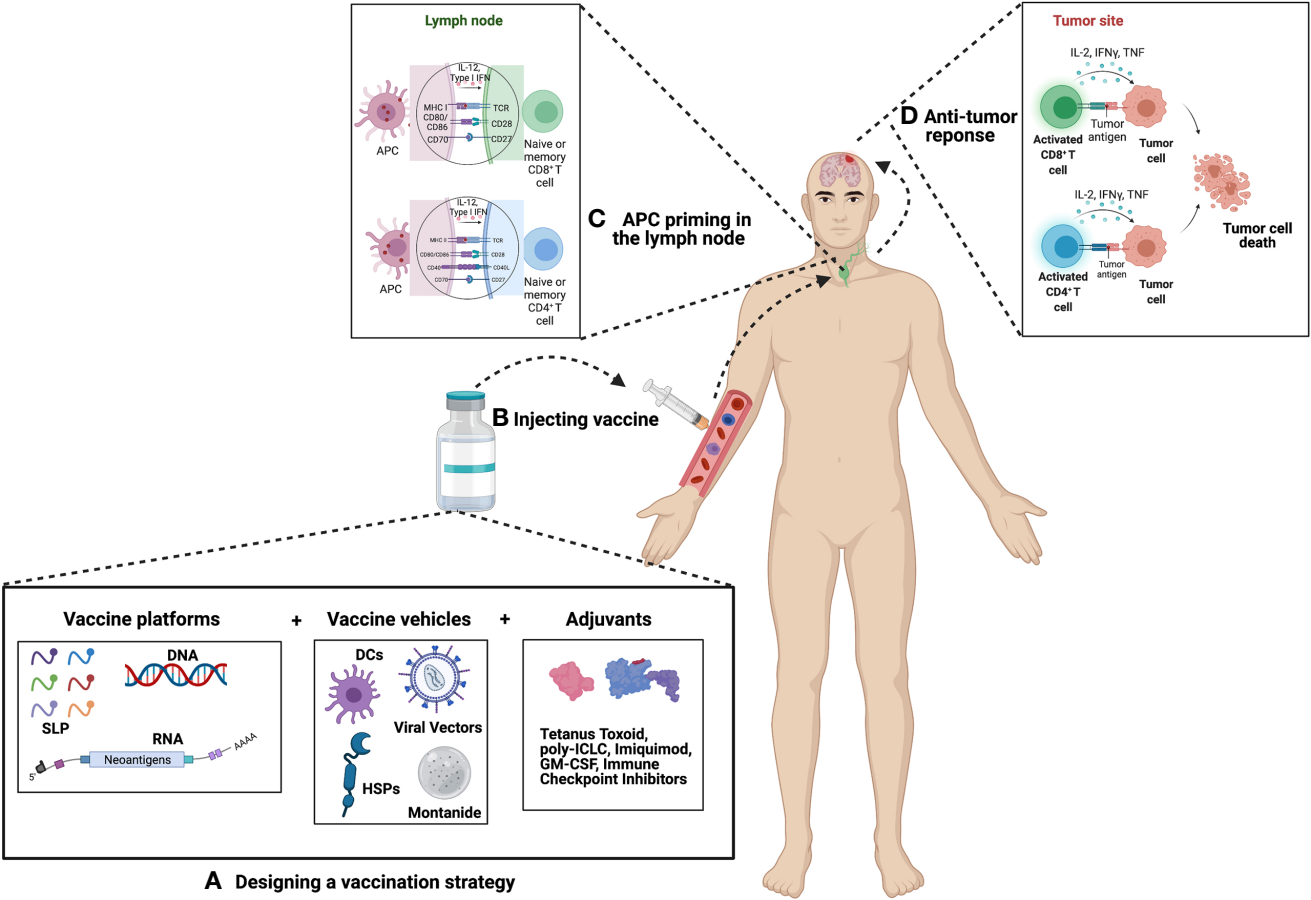
Principle of Cancer Vaccination. **(A)** Vaccine selection and preparation – Upon selection of suitable candidates a vaccine platform is chosen which includes either peptides, DNA or RNA. This platform is then packaged into a vehicle which includes either dendritic cells (DCs), viral vectors, heat shock proteins (HSPs), or montanide. The vaccine is then combined or paired with an adjuvant in an effort to boost the efficacy of the vaccine. Common choices of adjuvants include tetanus toxoid, poly-ICLC, imiquimod, GM-CSF, immune checkpoint inhibitors, as well as many others. **(B)** Vaccines can be administered intra-venously, intra-nodally, intra-dermally, or intra-muscularly. **(C)** Antigens are then presented by APCs to naïve or memory T cells in the lymph node. For GBM, presentation most commonly occurs in the deep-seated cervical lymph node. **(D)** Primed T cells migrate to the site of the tumor where they mount an anti-tumor immune response.

This review will highlight the most promising vaccines capable of treating GBM. In addition to discussing how these vaccines are made and their success in clinical trials, we will also explore pairing these vaccines with different adjuvants to enhance overall effect. Each vaccine trial will be categorized based on their target antigens, antigenic forms, vehicles used, as well as adjuvant pairings (see **Figure 1**). We hope that by highlighting the most promising vaccines and adjuvants, as well as discussing the need for robust immune monitoring in future clinical trials, this review can be used as a guide for designing novel vaccine-based approaches for treating GBM.

## Target Antigens

Antigen targets for vaccines are broadly classified as either tumor-associated or tumor-specific. Tumor-associated antigens are proteins expressed in many cells throughout the body in limited quantities, but are overexpressed in tumors (9, 19). Examples of these proteins in the case of GBM include survivin and Wilms tumor 1 (WT1). Tumor-specific antigens on the other hand include mutant proteins exclusively expressed by tumor cells (19). Examples include EGFRvIII and isocitrate dehydrogenase (IDH) R132H in the context of GBM and grade 4 astrocytoma, respectively (19). Generally, tumor-specific antigens are considered as ideal targets for a vaccine since they are selectively expressed on tumor cells and not in normal tissue. One of the challenges of GBM is the ability to find a tumor-specific antigen that is expressed uniformly within the tumor, is shared between patients, and is present after the widespread changes that occur with disease recurrence (1, 11).

Neoantigens are proteins that arise from mutations within a tumor cell and vary from cell to cell and person to person (20). Personalized neoantigen vaccines use sequencing data from the whole exome and RNA of a patient’s tumor to identify specific mutations particular to that individual (1). Most of these are “passenger mutations,” which derive from genomic instability within the tumor and do not play a role in tumorigenesis (20). The process of developing a neoantigen vaccine starts with DNA and RNA sequencing of the patient’s normal cells and the tumor (21). Analysis identifies mutational differences between the two, followed by RNA sequencing data which predicts the expression level of those mutations (21). MHC or human leukocyte antigen (HLA) typing is critical because the peptide that can be presented by MHC depends on MHC/HLA haplotype (21).

The activation of neoantigen-specific T cell responses requires T cell receptor recognition and binding to a specific epitope on the MHC. Upon transcription and translation of the neoantigen, the protein is cleaved into short peptide sequences that can be presented on class I MHC on tumor cells or class II MHC on antigen presenting cells (20). In the class I MHC pathway, intracellular protein fragments are transported into the endoplasmic reticulum *via* the TAP protein to be bound to MHC (21). Antigen presenting cells endocytose antigens that are cleaved by proteases in endosomes and then load these onto class II MHC (21). A large number of computational algorithms have been developed to predict the neoantigens that will undergo each step of this process and successfully lead to T cell activation (21). For example, predictions can be made to identify mutations that will lead to immunogenic neoantigens that are capable of binding to MHC molecules with high affinity (20). However, the prediction algorithm continues to be optimized and currently there is no standard (22).

Two phase I trials in 2019 tested the use of a personalized neoantigen vaccine strategy in newly diagnosed GBM patients (23, 24). The study by Keskin et al. (24) tested the approach in MGMT-unmethylated GBM patients after they had undergone surgery and standard radiation. Development of the vaccine first used whole-exome sequencing to compare data from the tumor samples to normal tissue. Specific single-nucleotide mutations were identified as candidates, RNA sequencing confirmed expression, and then predictions were made for the binding affinity of the neoantigens with the patient’s specific MHC/HLA alleles. The study enrolled 10 patients and the sequencing data identified a median of 116 somatic single-nucleotide mutations per tumor, which included genes such as *PTEN*, *EGFR*, and *RB1*. Interestingly, only patients who had not received dexamethasone during vaccine administration developed CD4^+^ and CD8^+^ T cell responses specific to the neoantigen of interest. These T cells could be detected in the peripheral blood, and the median PFS was 7.6 months alongside the median OS of 16.8 months. Unfortunately, each of the patients experienced relapse with progressive disease and the tumor-associated T cells showed an exhausted phenotype after vaccination. Thus, the authors noted that the therapy may be more effective in combination with ICIs.

A second study (GAPVAC-101) conducted by Hilf et al. (23) tested the concurrent administration of both a tumor-associated and a tumor-specific vaccine. APVAC1 was a tumor-associated vaccine with 5-10 unmutated peptides identified by expression profiling that most highly associated with the individual’s tumor (19, 23). APVAC2 was a personalized vaccine with 1-2 mutated neoepitopes (19, 23). The vaccines were given in conjunction with standard radiation and temozolomide and the authors concluded that administration of the vaccine with unmutated peptides led to prolonged central memory CD8^+^ T cell responses, while the personalized neoepitope vaccine primarily induced a Th1 CD4^+^ T cell response (23). The authors suggested that studies would be required to confirm these preliminary results (23).

These initial clinical trials have now led to two on-going clinical trials that combine a personalized neoantigen vaccine with immune checkpoint blockade. The first (NCT02287428) study is using a vaccine strategy that targets 20 mutant peptides directly expressed on the patient’s tumor (19, 25) in combination with pembrolizumab (anti-PD-1 antibody) and radiation therapy in newly diagnosed patients with MGMT-unmethylated GBM (25). This study divides patients into three different cohorts where vaccine and ICI is administered at different timepoints in relation to one another along with radiation therapy (25). This is important since it allows the study personnel to investigate if the timing of administration of pembrolizumab enhances efficacy of the vaccine (25). The second clinical trial (NCT03422094) uses a similar vaccine in newly diagnosed patients with MGMT-unmethylated GBM (26). However, the investigators in this trial combined treatment with nivolumab (yet another anti-PD-1 antibody) with the CTLA-4 antagonist, ipilimumab (26). Since anti-CTLA-4 and anti-PD-1 therapies have different mechanisms of action (27), this second trial will provide interesting insights with regard to the treatment of GBM.

## Vaccine Platforms

### Peptides

Peptide vaccines are composed of short chains of amino acids to induce activation of T cells. The presentation of these peptides by dendritic cells (DCs) in the draining lymph nodes prime antigen-specific T cells. Work done in human papillomavirus-associated cervical cancer first identified the enhanced efficacy of a 35 amino acid long-peptide vaccine (28). Longer peptides induced efficacious tumor immunity in mice and humans superior to minimal epitope peptides that fit MHC class I exactly because they were more likely to be processed and presented by professional antigen presenting cells, DCs (28, 29). Peptide vaccines are some of the most commonly used vaccines tested for the treatment of GBM and are composed of single or multiple antigens. Peptides tested as a single-antigen have included EGFRvIII, CMV pp65, TERT, IDH1, survivin, and WT1. These include epitopes of tumor-associated or GBM-specific antigens.

The GBM-specific EGFRvIII is a truncated mutant of the epidermal growth factor receptor (EGFR) (11, 30, 31). It is present in 20-30% of GBM patients and is expressed heterogeneously throughout the tumor (30, 31). The loss of exons 2-7 in the extracellular domain of the protein leads to the continuous activation of the growth factor signaling pathway (30). A peptide of 14 amino acids, which includes the novel epitope created by the deletion, was conjugated to keyhole limpet hemocyanin and formed the Rindopepimut vaccine (30). ACT IV was a multicenter phase III clinical trial that investigated the OS of patients receiving the Rindopepimut vaccine administered with temozolomide. This trial enrolled 745 patients with newly diagnosed GBM (11, 30). Despite the vaccine producing a notable humoral response, there was no significant survival benefit compared to control (30). The median OS of the Rindopepimut group was 20.1 months and the median OS of the control group was 20.0 months (11). The failure of this trial illustrates the limitation of the single antigen approach (11). EGFRvIII is expressed heterogeneously in 37-86% of tumor cells. Therefore, the successful induction of immune responses will allow the expansion of antigen negative tumor cells because they are not recognized by T cells activated by the vaccine, a process that is called immune selection (11, 32). In both arms of the study, around half of patients had loss of EGFRvIII expression upon recurrence (19). The randomized phase II ReACT trial explored the efficacy of Rindopepimut together with the anti-angiogenic bevacizumab in 72 patients with relapsed EGFRvIII-positive GBM (31, 33). PFS at 6 months favored the experimental group, suggesting that combination treatments may show promise despite previous monotherapy vaccine failures (31, 33).

Another potentially important antigen is cytomegalovirus (CMV) phosphoprotein 65 (pp65). Cytomegalovirus infects a large majority of adults and CMV proteins are expressed on greater than 90% of GBMs, with 50-70% of them positive for pp65 (34). Importantly, CMV proteins are suitable tumor-specific antigens since they are only found on GBMs and are not present on normal brain parenchyma (34). Preclinical studies have shown that CMV-reactive T cells effectively kill GBM cells positive for the pp65 antigen (19). A phase I trial is currently underway called PRiME (NCT03299309) which is testing the peptide vaccine PEP-CMV in malignant glioma and medulloblastoma patients (35). The therapy contains Component A which is a 26 amino acid peptide of the human pp65 CMV antigen and the study is actively recruiting patients ages 3-35 years old (35).

Two other tumor-specific antigens of note for single-antigen GBM vaccines include TERT and IDH1. Each plays an important role in the molecular classification of CNS tumors (36, 37). Promoter mutations of TERT (telomerase reverse transcriptase) are commonly present in GBM and expression of the protein is enhanced in many different cancer types (31). UCPVax is a peptide vaccine derived from TERT epitopes that induce Th1 CD4^+^ T cell responses (38, 39). A phase I/II clinical trial (NCT04280848) is currently evaluating this approach in GBM patients (38). Peptide vaccines for the IDH1 R132H mutation have also been developed for grade II and III gliomas (11). Around 80% of these low-grade tumors have an IDH mutation, of which the IDH1 R132H substitution is the most common (30). The benefit of this tumor-specific target is that it is present on every tumor cell (11). An IDH1 vaccine created in 2014 also had peptide that can be presented by class II MHC and induced a Th1 CD4^+^ T cell response (40). Two phase I clinical trials that have studied IDH1R132H peptide vaccines include NOA-16 (NCT02454634) and RESIST (NCT02193347) (41, 42). Each vaccine contains a peptide that includes the IDH1 R132H mutated sequence and administration was combined with temozolomide (41, 42). NOA-16 is completed and enrolled 33 patients with grade III and IV gliomas and the RESIST trial is still enrolling patients with grade II tumors (41–43). NOA-16 was shown to be safe and immunogenic with 93.3% of patients having IDH1 R132H-specific T cells (identified by ELISPOT or ELISA) that were not present before vaccination (43).

Tumor-associated antigens that have been tested in single-target GBM vaccines include survivin and WT1. Survinin prevents apoptosis in cells by inhibiting caspase activation and is highly expressed in GBM and other cancers (34). SurVaxM is a peptide vaccine of amino acids 53 through 67 of the protein, conjugated with keyhole limpet hemocyanin (44). A phase II study (NCT02455557) is currently investigating the treatment of 64 newly diagnosed GBM patients with temozolomide and the SurVaxM vaccine (34, 45). Early results indicate high titers of survivin antibodies and CD8 T cells after administration of the vaccine (46). The data also point to improvement in PFS and OS compared to historical controls (46). WT1 is a transcription factor, with DNA-binding that promotes oncogenesis (34). The peptide vaccine for WT1 has also shown to be effective inducing humoral and cytotoxic CD8^+^ T lymphocyte responses (47). A phase II study in 21 patients demonstrated a 9.5% clinical response rate and a PFS at 6 months of 33.3% (48). An additional peptide vaccine under investigation that induces WT1-specific T cell responses is DSP-7888 (49). It has been tested in multiple types of advanced malignancies (NCT02498665) (50), pediatric high grade glioma (NCT02750891) (51), and in combination with bevacizumab for the treatment of recurrent or progressive GBM (NCT03149003) (52). Importantly, another clinical trial (NCT03311334) is underway in other solid tumors that combines DSP-7888 with immune checkpoint inhibition (53).

Peptide vaccines have also been developed that include multiple antigens. One such example combined three tumor-associated antigens overexpressed in childhood gliomas (survivin, IL-13 receptor alpha 2, and EphA2) (54). Preliminary evidence with enzyme-linked immunosorbent spot analysis showed that 13 of 21 patients mounted positive responses to at least one of the antigens (54). IMA950 is a vaccine with 11 different tumor-associated antigens (one of which is survivin) and each antigen was found present on HLA in GBM tissue samples (55). Nine of the peptides bound class I MHC, two bound class II MHC, and each was chosen based on ability to activate CD4^+^ and CD8^+^ T cells (55). A phase I/II study tested the vaccine in newly diagnosed GBM patients which found that it elicited CD8^+^ and Th1 CD4^+^ T cell responses and led to a median survival of 19 months (56). A current clinical trial (NCT03665545) is combining the IMA950 vaccine with pembrolizumab (57). Strategies such as this with multiple antigens or combinatorial approaches will be necessary to outcompete the heterogeneity and immunosuppression of GBM.

### Nucleic Acids

The development of DNA vaccines is a recent strategy that is being tested in patients with GBM. Bacterial DNA plasmids that encode tumor-associated antigens and immune-stimulating cytokines are inserted into host cells, thus enhancing the expression of these molecules (58). One major benefit of DNA vaccines is that once the plasmid is in the nucleus, the antigens can be presented on both class I and class II MHC (58). The expressed antigens can activate the normal cytotoxic CD8^+^ T lymphocyte and Th1 CD4^+^ T cell responses that normally play a role in combating intracellular pathogens or malignancies (58, 59). The technique also activates the innate immune response through the recognition of bacterial CpG motifs and double-stranded DNA-sensing receptors (58, 59). Electroporation, a commonly used method for plasmid delivery into the nucleus of host cells, delivers brief, high intensity electricity to induce increased membrane permeability (59). The process also has a pro-inflammatory benefit with the release of cytokines that increase immune cell concentrations to the site of delivery (59). Recent progress in the field of DNA vaccines has drastically increased their efficacy by optimizing the codons used and untranslated RNA transport elements (60, 61). A phase I/II clinical trial (NCT03491683) is currently investigating the efficacy of the DNA vaccines INO-5401 and INO-9012 combined with the PD-1 antagonist cemiplimab in newly diagnosed GBM patients (62, 63). INO-5401 expresses the tumor-associated antigens WT1, PSMA (prostate specific membrane antigen), and TERT, while INO-9012 encodes the p35 and p40 subunits of IL-12 (62, 63). Both of the vaccines are administered with an intramuscular injection with subsequent electroporation (62, 63). The study is still ongoing, but interim analysis identified that the therapy is safe, immunologically effective, and may lead to an encouraging survival advantage (64). A similar DNA vaccine phase I trial (NCT04015700) with 6 participants is also underway using a personalized neoantigen DNA vaccine, INO-9012, and electroporation (65).

RNA vaccines are in the early stages of development as a potential treatment of GBM patients. The idea behind this approach is that a desired mRNA can be injected in the form of a vaccine and the subsequent proteins are expressed in the cells of the patient (66, 67). The mRNA will encode the antigen of interest, 5’ and 3’ untranslated regions, a 5’ cap, and a poly A tail (66). Translation occurs in the cytosol without the need for transport to a specific organelle, and then normal degradation decreases the chance for toxicity (66). Only recently have protocols been developed which have allowed the stable, efficient delivery of mRNA *in vivo* (66, 67). Many of the benefits of the mRNA approach over other vaccines include its safety and manufacturing (66). Messenger RNA is not an infectious agent, will not insert into the human DNA genome, and manufacturing can be quickly and inexpensively increased (66). Downsides of RNA vaccines continue to be storage and lifetime, but efforts are being made to combat these problems (66). A phase I/II RNA vaccine study (NCT04573140) is underway in GBM patients in the form of lipid particles that are loaded with the mRNA (68). Given the benefits of RNA vaccines, it is anticipated that the number of GBM vaccine trials with this approach will continue to increase.

## Vaccine Vehicles

### Dendritic Cells

DC are professional antigen presenting cells (APCs) with the ability to capture and present exogenous antigens (69). The ability of DCs to stimulate CD8^+^ T cells with peripheral antigens *via* class I MHC molecules makes them ideal vehicles for administering GBM vaccines. DCs loaded with glioma antigens *ex vivo* can be administered to patients for activation of T cells and induction of robust cytotoxic activity (69). Antigen-loaded DCs must migrate to the lymphoid organs to activate T cells (69). Once activated, T cells that successfully traffic to the tumor site can exert cytotoxic effects on antigen-expressing tumor cells, provided that the tumor microenvironment (TME) is not overly immunosuppressive.

Though classical DCs are undetected in healthy brain parenchyma, they are present in proximal vascular-rich tissues including the choroid plexus and meninges (70). Additionally, in pathological conditions, DCs are capable of migrating to the brain through the afferent lymphatics or the high endothelial venules, and are readily recruited to parenchymal inflammatory lesions (69, 70). This suggests that DCs are capable of recognizing and presenting brain-derived antigens in order to stimulate effector T cells to combat brain tumors. However, compared to other organs, drainage of brain tumor antigens is inefficient and trafficking of immune cells to the brain is attenuated. Viewed optimistically, the native limitations of CNS DCs indicate great potential for therapeutic interventions capable of promoting DC-mediated presentation of glioma antigens to peripheral T cells.

The current generation of DC vaccines are derived from specific subsets of freshly isolated, patient-derived DCs from peripheral blood cultured *ex vivo* with a maturation cocktail of proinflammatory cytokines such as PGE1, TNF-alpha, and IL-1beta (70, 71). Before administration to patients, DC vaccines are pulsed with antigens from a variety of sources, including peptides, tumor lysates, tumor RNA, vectors expressing tumor-associated antigens, and tumor-derived exosomes (69, 70). The most common route of DC vaccine administration in GBM patients has been intradermal, although intravenous, intranodal and intramuscular routes are also possible (69, 70). Though autoimmune reactions caused by DC vaccines are a potential concern, DC vaccines have demonstrated minimal to low toxicity in over 10 phase I/II trials in GBM patients (30).

DCs as vehicles for administration of GBM antigens have been explored in a variety of clinical trials. The first major category of DC vaccines are those expressing single tumor antigens, with most in early stages of clinical investigation. A phase I trial of newly diagnosed GBM patients receiving DCs pulsed with EGFRvIII conjugated to keyhole limpet hemocyanin was demonstrated to be immunogenic in 10 of 12 patients with no serious adverse events (72). A phase I trial in which patients with recurrent glioma received DCs pulsed with WT1 also reported no serious adverse events, and 6 of 10 patients showed a two-fold or greater increase in WT1-specific cytotoxic T lymphocytes by tetramer analysis (73). The phase I/II ADDIT-GLIO trial (NCT02649582) is currently investigating the effectiveness of autologous WT1 mRNA-loaded DCs in combination with TMZ (74). The ICT-121 vaccine targets the cancer stem cell antigen CD133 and is comprised of autologous DCs loaded with two HLA-A2 restricted CD133 epitopes (75). ICT-121 was demonstrated to be safe and to generate immune responses in a phase I trial of patients with recurrent GBM (75). A small phase I trial of patients with recurrent glioma also demonstrated safety and immunogenicity of DCs pulsed with IL-13 receptor alpha 2-derived peptides (76).

In addition to these studies, a number of trials have investigated the usage of DCs pulsed with mRNA encoding the immunodominant CMV pp65 antigen (77, 78). Pooling results from multiple trials utilizing CMV pp65 DC vaccines, it was recently reported that nearly a third of patients receiving treatment have survived beyond 5 years, indicating high promise for these treatments (79). A phase II clinical trial investigating TMZ plus CMV pp65-LAMP mRNA-pulsed DCs administered with GM-CSF and tetanus-diphtheria toxoid is ongoing in patients with newly diagnosed GBM (NCT02465268) (80). Additionally, the Phase I AVERT study (NCT02529072) of pp65-LAMP mRNA-pulsed DCs combined with nivolumab has been completed in patients with recurrent gliomas and additional phase II studies are anticipated (81).

A benefit of using DC vaccines over therapies, such as adoptive transfer of chimeric antigen receptor (CAR) T cells is that they can be used to generate responses to a multiplicity of antigens. The second major category of DC vaccines are pulsed with multiple selected antigens, creating an opportunity to activate CD8^+^ T cells specific for a variety of targets, which may be a beneficial strategy for combating the heterogeneity of GBM. Ideally, DCs pulsed with multiple common glioma antigens, such as WT1, EGFRvIII, and survivin, could serve as “off-the-shelf” therapies capable of treating a variety of GBM patients (69). However, a downside of this approach is the potential misallocation of immune “resources,” (*i.e.* generation of activated T cells specific for antigens not actually expressed on a particular patient’s tumor) as this may dilute the effects of vaccination against expressed antigens (70).

The most extensively studied multi-peptide pulsed DC vaccine is ICT-107, which consists of autologous DCs pulsed with six synthetic peptides: HLA-A1-restricted melanoma-associated antigen-1 (MAGE-1) and antigen isolated from immunoselected melanoma-2 (AIM-2), as well as HLA-A2-restricted human EGFR-2 (Her2/neu), tyrosine-related protein-2 (TRP-2), glycoprotein 100 (gp100), and IL-13 receptor alpha 2 (82). In a randomized phase II trial of newly diagnosed HLA-A1^+^ and/or HLA-A2^+^ patients receiving the ICT-107 vaccine, no significant difference in OS was observed in the treatment group as compared to controls (82). However, PFS significantly favored the treatment group by 2.2 months. Additional analyses revealed that while over 90% of patients expressed all the HLA-A2 antigens, only 38% of patients expressed the HLA-A1 antigens, and for HLA-A2^+^ patients with a methylated MGMT promoter, median PFS was 24.1 months for the ICT-107 treatment group compared to a median PFS of 8.5 months for the controls (82). A phase III trial of ICT-107 plus TMZ restricted to HLA-A2^+^ GBM patients was underway but has been suspended due to a lack of funding (31).

A third approach to DC vaccination involves pulsing DCs with autologous whole-tumor lysate. This class of DC vaccines has been the most extensively studied to date and offers the advantage of being personalized to each patient’s unique tumor profile. It also allows for presentation of a comprehensive repertoire of heterogeneously expressed TAAs and neoantigens without a need for identifying them (70). However, such indiscriminate antigen presentation may be capable of driving extraneous or even harmful responses against non-tumor antigens, though a large number of clinical trials have demonstrated minimal toxicity of this approach (69).

DC-VaxL is a tumor-lysate pulsed DC vaccine and is the only phase III DC vaccine trial with published interim results at this time (83). At the interim analysis, the median OS for the intent-to-treat population was 23.1 months from surgery, with 46.6% of patients with methylated MGMT surviving three years (83). While this data appears exciting, the unblinded survival data and immunological results remain highly anticipated. However, a different tumor-lysate-pulsed DC vaccine (Audencel) evaluated in a randomized, controlled phase II study of patients with newly diagnosed GBM (GBM-Vax) showed no significant difference in OS between the treatment and control groups (84).

There are also a number of non-controlled phase II trials of tumor-lysate pulsed DCs with or without temozolomide that have been completed in patients with *de novo* GBM. These trials have shown immunoreactivity in 25-40% of patients (where reported) and patient OS ranging between 18.3 to 28 months (85–88), with MGMT methylated patients showing a median OS of 32.8 months in one study (88). In a phase II study of 23 patients with recurrent GBM and 11 patients with newly diagnosed GBM receiving tumor lysate-pulsed DCs, 50% of patients had positive vaccine responses as indicated by a 1.5 or more fold enhancement of IFN-gamma production compared to pre-vaccination levels. Vaccine responders had significantly longer median OS compared to non-responders (642 vs. 430 days) (89). A number of additional phase I and II clinical trials involving autologous tumor-lysate pulsed DCs in GBM are currently ongoing, including phase I trials investigating new adjuvant therapies such as topical imiquimod, cyclophosphamide + nivolumab/ipilimumab, and pembrolizumab + Poly-ICLC (NCT01808820) (NCT03879512) (NCT04201873) (31).

Another approach to DC vaccination gaining interest in recent years involves pulsing DCs with glioma stem cells (GSC) components. In a phase I clinical trial of 7 GBM patients receiving DCs pulsed with mRNA-derived from autologous GSC cultures, PFS was 1.9 years and increased lymphocyte proliferation in response to GSC lysate exposure *in vivo* was observed in all 3 patients with testable material (90). Additional trials involving GSC DC vaccines are ongoing (NCT01567202) (NCT02010606) (NCT02820584).

A recent meta-analysis of randomized controlled studies using GBM DC vaccines demonstrated that DC vaccination was associated with significantly improved overall survival in GBM patients (91). However, only six studies were included in this analysis due to strict inclusion criteria and the lack of randomized, controlled studies. This highlights the need for larger, thoughtfully-designed studies evaluating DC vaccine efficacy. Further research into the optimization of DC vaccines, including optimal adjuvant strategy, tumor antigens, pulsing scheme, and combinatorial treatments are needed.

Because the success of DC vaccines ultimately lies in the ability of DC-activated T cells to successfully exert cytotoxic effects, it is important that the GBM microenvironment does not suppress CD8^+^ T cell activity. In this context, the combination of DC vaccines with checkpoint inhibitors such as nivolumab warrants more thorough investigation. Checkpoint inhibitors have the ability to combat T cell exhaustion, thus facilitating more effective T cell mediated anti-tumor lytic activity. The immunosuppressive microenvironment of gliomas may greatly hamper the impact of a DC vaccine in the absence of combinatorial therapies, and may be the reason that DC vaccines have had limited success in clinical trials thus far.

### Heat Shock Proteins

Heat shock proteins (HSP) are critical in cell survival as the production of these proteins becomes upregulated whenever a cell is undergoing a stressful event. These stressful conditions can range from the cell being too hot or cold, undergoing UV radiation, having an osmolarity that is too high or low, or an abnormal acid-base status (92). HSPs were originally discovered by observing cells that were overheated, hence how the name “heat shock” originally came about. Once a cell undergoes stressful event, this can either result in the halting of protein production, or more commonly the misfolding of proteins. These misfolded proteins then begin to aggregate within the cell, which can eventually lead to cell death. The cell employs HSPs to prevent these deleterious events from happening by limiting the number of misfolded proteins within the cell in two different ways. If the misfolded protein can be refolded so that the protein gains functionality, the HSP will serve as a chaperone and bind to hydrophobic regions of the misfolded protein to help it fold properly (92). If the HSP cannot refold a misfolded protein due to significant misfolding, the HSP will assist in degradation by shuttling the protein to the proteosome.

HSPs are of great interest to the oncology community because their production is upregulated in cancer patients as tumors have an increased expression of misfolded or abnormal protein products. To avoid cell death as a result of an aggregation of misfolded proteins, it is believed that tumors increase the production of HSPs (93). In patients with GBM specifically, it has been reported throughout the literature that these patients have an increased expression of HSP27, HSP72, HSP73, and HSP90 (93, 94). It has also been reported that HSP27, HSP60, HSP70, and HSP90 are present within exosomes released by GBM tumors (93, 95). However, using HSPs alone to prime the immune system in order to evoke an anti-tumor immune response would not be successful as a vaccine platform as HSPs alone are unable to evoke immune responses. Alternatively, when HSPs and peptides are brought together into complexes (HSPPCs), these can elicit class I MHC -based CD8^+^ cytotoxic T lymphocyte responses (96). This is important because exogenous antigens are typically presented by class II MHC molecules leading to CD4^+^ T helper cell responses, yet HSPPCs induce robust CD8^+^ T cell responses (97). The key to having these HSPPC-derived peptides presented on class I MHC molecules is the CD91 receptor on antigen presenting cells, which allows for the uptake of HSPPCs into the cell (97). Once these complexes are inside the cell, they will ultimately be broken down *via* proteosomes, and then shuttled to the endoplasmic reticulum to be loaded onto class I MHC molecules (98). While the majority of the internalized protein follows the pathway mentioned prior, it is also important to note that some of the internalized HSPPC can be loaded into an acidic compartment which allows for loading onto class II MHC (93). This finding is pivotal as it shows that HSPPCs can stimulate both CD8^+^ and CD4^+^ T cells, a major benefit for using HSPPCs in anti-cancer vaccines.

HSPPCs can also interact with a variety of receptors that allow for activation of the NF-κB pathway (93). Additionally, it has been observed in macrophages that these HSPPCs can upregulate the secretion of pro-inflammatory cytokines such as TNF-alpha as well as IL-12 (99). Given that HSPPCs are capable of inducing pro-inflammatory responses in multiple different ways, it is clear why they are being used in vaccines. HSPPCs are capable of providing more than one antigen for presentation (100). Given the heterogeneity of tumors such as GBM, having a vaccine which accounts for more than one antigenic target is a far more improved approach.

The vast majority of HSP vaccines have used HSPPC-96 because of observed safety and minimal toxicity. The HSPPC-96 vaccine is created by isolating HSPs from patient tumor specimens. The HSPs are expected to be bound with proteins including tumor antigens made by tumor cells. Once enrichment is complete, the purified HSPs are given to patients on a weekly schedule for the first month and then on a bi-weekly schedule until the vaccine supply has been fully depleted (101). Overall, the vaccine has been well tolerated by patients with GBM in phase I trials. In a phase II trial that enrolled patients with recurrent GBM, 90.2% of patients receiving the HSPPC-96 vaccine were alive at 6 months following treatment, whereas 29.3% of patients receiving the HSPPC-96 vaccine were alive at 12 months following treatment. The median OS for patients receiving the HSPPC-96 vaccine was 42.6 weeks (93, 102). An exciting trial that is currently ongoing (NCT03018288) is treating newly diagnosed GBM with radiation therapy and temozolomide while combining pembrolizumab with or without HSPPC-96 (103). One of the goals of this trial is to determine if combining pembrolizumab with HSPPC-96 provides a synergistic effect. This is being compared to the immune response of patients receiving only radiation therapy, temozolomide, and pembrolizumab (103). Clinical trials have demonstrated that HSPPC vaccines promote a survival benefit in patients with GBM. While this may appear promising, far more clinical trials are needed to determine if pairing this vaccine platform with different adjuvants such as ICIs will promote long-term survival in patients with GBM.

## Adjuvants

Unsuccessful vaccine trials in GBM are thought, in large part, the result of the intense immunosuppression caused by the disease (19). A combination of vaccines paired with adjuvants may be able to overcome these immunosuppresive mechanisms (19). Adjuvants are given in addition to the vaccine to enhance the immune response to a particular antigen (30). This is accomplished by either promoting the ideal presentation of the antigen, inducing the expression of co-stimulatory molecules, or prompting the release of cytokines by antigen presenting cells (104). The most successful and commonly used adjuvants in GBM vaccine trials include montanide, tetanus toxoid, poly-ICLC, imiquimod, CpG nucleotides, and GM-CSF. It is anticipated that the success seen with ICIs in other cancers will also translate over to GBM when used as a vaccine adjuvant.

Montanide is the clinical-grade of Incomplete Freund’s Adjuvant (Complete Freund’s Adjuvant without the *Mycobateria tuberculosis*) (104). As a water-in-oil emulsion, the adjuvant enhances the length of antigen presentation by retaining and slowly releasing the antigen at the site of vaccination (104). Two preparations of Montanide used as an adjuvant in human vaccine trials include Montanide ISA 51 and Montanide ISA 720 (105). Each uses a mannide monooleate surfactant, the difference being that Montanide ISA 51 uses a mineral oil and Montanide 720 a nonmineral vegetable oil (105).

In 2003 it was noted that the tetanus-diphtheria toxoid could improve the efficacy of DC vaccines in GBM patients (78). Mitchell et al. primed the vaccine site with a dose of the toxoid prior to vaccinating with CMV pp65-pulsed DCs (78). This significantly improved DC migration to lymph nodes and improved OS and PFS (78). Thus, two current clinical trials (NCT02366728) and (NCT03927222) are studying this pre-conditioning technique with the tetanus-diphtheria toxoid in the context of the CMV pp65 DC vaccine (106, 107).

Poly-ICLC, Imiquimod, and CpG oligonucleotides each activate the innate immune system by binding and activating toll-like receptors (TLRs): poly-ICLC to TLR3, Imiquimod to TLR7/8, and CpG to TLR9 (104). Poly-ICLC, also known as Hiltonol, is a stable double-stranded RNA derivative of poly I:C (polyinosine-polycytidylic acid) (104). Imiquimod is a synthetic imidazoquinoline that mostly activates TLR7, while resiquimod acts on TLR7 and TLR8 (104). TLRs 7 and 8 are each activated by single-stranded RNA and upregulate costimulatory molecules (CD80/86 and CD40), increase cytokine production (IFN-alpha, TNF-alpha, and IL-12), and enhance lymph node DC migration (104). An active phase II clinical trial (NCT01204684) is comparing the efficacy of imiquimod/resiquimod versus poly-ICLC in a tumor-lysate pulsed autologous DC vaccine (108). Lastly, TLR9 is activated by unmethylated CpG nucleotides, a pathogen-associated molecular pattern indicative of bacterial DNA (104, 109). These CpG nucleotides stimulate professional antigen presenting cells such as B cells and DCs leading to Th1-specific responses (109). These innate system agonists may prove pivotal in the challenge to surmount the multiple mechanisms of immunosuppression in GBM.

GM-CSF, or granulocyte-macrophage colony stimulating factor, is a cytokine growth factor that stimulates the activity and enhances the production of neutrophils, monocytes, and eosinophils (104, 110). Vaccine studies have shown that the adjuvant leads to DC maturation and recruitment, and macrophage, NK cell, and neutrophil activation (104). GM-CSF can not only be used as a recombinant protein, but also expressed by transfected tumor cells in a vaccine known as GVAX (111). To date, no clinical trial has used GVAX in GBM.

ICIs provide a promising approach for general activation of the immune system (19). CTLA-4 and PD-1 are both negative regulators of immune cell function (27). CTLA-4 acts early in the immune response in lymphoid tissues by preventing the binding of B7 on the antigen presenting cell to the T cell costimulatory molecule CD28 (27). PD-1 on T cells acts later in the peripheral tissue by initiating an inhibitory signal after binding to PD-L1 on tumor cells (27). ICIs are not limited to PD-1, CTLA-4, and PD-L1. Studies have shown promise with the antagonism of TIM3, LAG3, and VISTA (32, 112). Another possible inhibitory receptor to target is TIGIT (113). In addition, approaches to agonize molecules that activate T cells have also been used. The costimulatory molecule OX40 (CD134) is a part of the tumor necrosis factor superfamily and binds with the OX40 ligand (CD252) on antigen presenting cells (114). Expression is only present after antigen stimulation, thus OX40 co-stimulation is a late signal to enhance effector T cell survival (114). A preclinical study combined an OX40 agonist with an irradiated GL261 tumor cell GVAX vaccine (115). The result was increased survival by 14 days compared to controls, as well as Th1 responses and CD8 to T regulatory cell ratio (115). The authors also noted that combination therapy improved T cell exhaustion phenotypes with decreased expression of PD-1, TIM-3, and LAG-3 (115). An additional costimulatory molecule that has been studied in GBM vaccine trials is CD27. The monoclonal antibody varlilumab is an agonist of CD27, mimicking the physiological interaction with CD70 on antigen presenting cells which initiates T cell proliferation and activation (116). The ongoing DERIVe clinical trial (NCT03688178) is investigating a CMV pp65 DC vaccine together with varlilumab (117). The study will continue the previously discussed work of pre-conditioning by comparing groups treated with the tetanus-diphtheria toxoid and control prior to administering the vaccination (117).

A final immune checkpoint target that has shown efficacy in preclinical models of GBM treatment is the CD47-SIRP-alpha axis. CD47 is an antiphagocytic transmembrane protein that is upregulated on tumor cells to initiate immune escape (118). CD47 binding to the inhibitory signal regulatory protein-alpha (SIRP-alpha) on myeloid cells initiates a “don’t eat me” signal and prevents macrophage phagocytosis (119). Hu5F9-G4 is a humanized anti-CD47 antibody that showed clinical efficacy in mouse xenograft models of patient-derived pediatric brain tumors (119). Inhibition of the CD47-SIRP-alpha axis in combination with autophagy inhibitors increased macrophage infiltration, tumor cell apoptosis, and median survival in mouse models of GBM (118). Despite promising preclinical results, blockade of this checkpoint target has yet to be tested in a clinical trial for GBM. Anti-CD47 treatment has shown to play an important role in enhancing macrophage phagocytosis of GBM and promoting an anti-tumor phenotype (120). Considering that tumor-associated macrophages are one of the major players of GBM mediated immunosuppression, clincal trials combining vaccination with an anti-CD47 adjuvant could prove to be quite efficacious.

ICIs have had broad success in many cancer types, thus it might be the most promising adjuvant to use in combination with vaccines. It is important to note that while the vast majority of research is focused around PD-1, PD-L1, and CTLA-4, the immune checkpoint repertoire is not restricted to this small subset. Trials into the future can continue to investigate proven strategies such as agonists for the innate immune system, or one of the novel immune checkpoints such as OX40 or CD27.

## Clinical Trials

Unfortunately, no vaccine targeted to GBM has met primary endpoints in a phase III clinical trial. The failure of these clinical studies, modeled after marked success of the approaches used in pre-clinical settings supports a re-evaluation of the clinical trial designs used to test immunotherapy in brain tumors. In most clinical trials for GBM there is a lack of robust immune monitoring. Rather, many of these trials evaluate only for OS and PFS and if the trial does not meet its endpoint for one of these indicators, the intervention is often labeled a failure. The cause for why the intervention failed is often not known. For example, in the ACT IV Rindopepimut clinical trial, the patients receiving the vaccine did not experience a survival benefit and hence the study was deemed unsuccessful. Retrospectively, it was identified that the patients did experience an enhancement of anti-EGFRvIII antibody titers as a result of the vaccine, indicating that the intervention performed its desired biological function but this was not sufficient to impact survival (121).

This finding reinforces the concept that GBM is both a heterogeneous and immunosuppressive disease thereby decreasing the likelihood that one intervention can target a sufficient number of cancer cells to result in a long-term survival benefit. Future treatment protocols for patients with GBM will most likely involve patients receiving a cocktail therapy targeted to multiple aspects of the tumor. However, evaluation of immunotherapeutic strategies must go beyond just determining the efficacy of an intervention by measuring OS or PFS. Robust immune monitoring must be incorporated into the design of clinical trials enabling the identification of interventions that enhance anti-tumor immunity. Frequent and longitudinal evaluations will help determine optimal timing of interventions and the duration of the immune response.

Immune monitoring can be incorporated into clinical trials using clinical imaging, blood correlative studies, and tissue analysis. Imaging studies are often capable of showing whether patients are experiencing a response to the intervention. However, imaging in brain tumor studies can be complicated as it can be quite difficult to distinguish between tumor progression and response to therapy (pseudo-progression) (122). Therefore, incorporating other imaging studies such as positron emission tomography (PET) into brain tumor immunotherapy clinical trials may be complementary and help confirm patient response to treatment (123–125). Radiomics is an additional imaging technique that may be advantageous to incorporate into clinical studies when evaluating for patient response to immunotherapy. This technique can take sets of clinical images and use computer algorithms to analyze differences in tumor shape as well as spatial orientation and structure (126). The results of this analysis can then be used to inform clinical teams about prognosis as well as whether the disease is progressing. While this technique has been used in studies to predict OS for patients with GBM, coupling radiomics with machine learning is needed to provide an objective indicator to differentiate between tumor progression and patient response to treatment (126).

Measuring patient response using peripheral blood is promising. Most T cell activation takes place in the periphery, when naïve T cells interact with APCs that present tumor antigens. Subsequently these T cells migrate to the site of the tumor in the brain where they may be subject to additional stimulation or suppression from the tumor cells or other factors in the TME. Therefore, it is likely that GBM patients who demonstrate a response to immunotherapy, first display a systemic effect. Activation of peripheral T cells would suggest a response to the intervention. Previous studies have shown that looking at peripheral markers of immune response such as the clonal expansion of T cells, expression of specific chemokine receptors, and levels of IFN-gamma can potentially determine whether a patient is experiencing a response to immunotherapy (122). In patients with GBM, it has been observed that an enhanced expression of IFN-gamma has been associated with better patient outcomes, while the IL-6 axis specifically has been associated with both increased tumor growth and expression of an M2-like myeloid phenotype (32, 122, 127). If patients experience an immune response as shown by markers within the peripheral blood yet fail to meet the primary endpoints of a trial, additional treatments may be needed to enhance either the trafficking of peripheral immune cells to the brain tumor or suppress the hostile TME.

Patient response to immunotherapy can also be evaluated through analyzing different expression levels of intra-tumoral markers. Specifically, intra-tumoral TCR diversity and clonality can be used. Cloughesy and colleagues observed that baseline increases in the TCR repertoire in patients with GBM may be associated with a survival benefit (128). This finding is new to the field as most studies have focused on how increased clonal size of T cells may promote a survival benefit as opposed to an increase in the overall TCR repertoire. Whether an increase in the TCR repertoire promotes a survival benefit in patients with GBM is still up for debate, however studies by Li and colleagues (who treated patients with a HSPPC-96 vaccine) observed the opposite (129). In fact, they noted that long term survivors with GBM expressed a lower amount of TCR diversity and a higher amount of TCR clonal expansion (129). While the benefit that TCR repertoire expansion provides to patients is still debated, clinical trials such as this have demonstrated that increased TCR clonal expansion promotes a survival benefit in patients with GBM. Evaluating for increased intra-tumoral TCR clonal expansion in patients with GBM may be worthwhile to help research teams understand whether patients are experiencing robust intra-tumoral immune responses to the intervention they are receiving.

In addition to incorporating robust immune monitoring into clinical trials of the future, there is a clear need for limiting the administration of immunosuppressive corticosteroids to patients enrolled in brain tumor clinical trials. Dexamethasone (a type of corticosteroid) is commonly given to newly diagnosed brain tumor patients in order to alleviate cerebral edema. However, it has been well documented by our group that dexamethasone upregulates the presence of CTLA-4, as well as blocks CD28-mediated cell cycle entry and naïve T cell differentiation (130). This results in an overall decrease in naïve T cell proliferation and differentiation. However, in pre-clinical models of GBM, it has been seen that treatment with anti-CTLA-4 or stimulation of T cells with strong activators such as CD28, prior to dexamethasone exposure can rescue T cells from the detrimental effects of this corticosteroid (130). A study by Reardon and colleagues demonstrated that dexamethasone administration concurrent with anti-PD-1 therapy reduced the survival of tumor-bearing mice in a dose dependent manner (131). It was also observed in this study that dexamethasone enabled an overall decrease in the number of T-cells as a result of increased T cell apoptosis (131). Lymphocytes that did not undergo apoptosis displayed a significant decrease in their overall function (131). The authors also note that dexamethasone reduced the number of myeloid and natural killer cell populations as well. While the studies showing the negative impacts dexamethasone has for brain tumor immunotherapy patients are still limited at this time, it may be worth considering using alternatives to dexamethasone in brain tumor immunotherapy trials of the future as dexamethasone may impact outcomes for brain tumor patients receiving immunotherapy.

One possible alternative to dexamethasone that should be considered for clinical trials of the future is mannitol. Mannitol is a sugar alcohol that reduces cerebral edema by creating an osmotic gradient within the brain that allows for movement of water from the parenchyma to the intravascular space which allows for a reduction in brain tissue volume as well as a lowering of intracranial pressure (132). While mannitol can decrease brain edema without causing immune suppression, it has been noted that increased doses of mannitol can lead to adverse events which is why more clinical trials are needed to determine the safety in using mannitol to manage edema in brain tumor patients (132). However, only a limited number of patients suffering from cerebral edema may benefit from receiving mannitol. It has been noted in the literature that using mannitol is problematic when treating patients with chronic cerebral edema (133). In addition to mannitol requiring IV infusion when treating chronic edema, mannitol diffuses into the brain over time which limits the effectiveness of this approach (133, 134). While mannitol may be effective in treating some patients with cerebral edema, its time-limited efficacy restricts use for short term or acute situations.

Bevacizumab has been proposed as an alternative to dexamethasone for combatting cerebral edema as it specifically targets vascular endothelial growth factor A (VEGF-A) which promotes both angiogenesis and vascular permeability (135). This finding suggests that VEGF-A plays a critical role in the increased brain edema that has been observed in patients with brain tumors (135). Bevacizumab sequesters VEGF-A thereby preventing binding to its receptors which allows for a reduction in cerebral edema as observed by Xiangying and colleagues (135) but does not extend survival in patients with GBM (136). However, with a prolonged plasma half-life and sustained inhibition of wound healing, the use of bevacizumab, while effective in reducing cerebral edema in patients with brain tumors, raises concerns in this patient population. Specifically, Bota and colleagues found that the optimum time for patients to stop receiving bevacizumab prior to tumor resection was four weeks (137). Additionally, the research team found that patients should not undergo treatment with bevacizumab for at least two weeks following surgery (137). The cessation of bevacizumab prior to surgery and wait time prior to re-initiating this treatment following surgery is necessary for patients to avoid surgical complications, thereby limiting widespread use of this agent as a substitute for corticosteroids.

Control of tumor-related cerebral edema remains a challenge. In the context of clinical trials, alternatives to corticosteroid use such as short-term mannitol and bevacizumab can be prospectively evaluated so that guidelines can be established to enable testing of immune therapies in this patient population where response is not further compromised by iatrogenic factors.

## Discussion

In this review, some of the most promising vaccines that are currently under investigation for the treatment of GBM were discussed including the rationale for their use and the clinical trial results thus far (see **Table 1**). Additionaly strategies such as the use of adjuvants and the importance of immune monitoring thereby enhancing the information obtained from clinical trials were also discussed. Hopefully, this review serves as a guide or provides an outline for investigators and clinicians as they seek to design and implement different vaccine-based approaches to treat patients suffering from GBM. While many vaccines targeted to GBM can stimulate the immune system, the benefit of this stimulation is often transient, failing to be sufficient enough to increase OS and/or PFS. The heavily immunosuppressive nature of GBM contributes to the failure for most immunotherapeutic strategies as does the heterogenous nature of GBM. Targeting multiple antigens, perhaps some commonly occurring in most GBMs such as EGFRvIII, IL-13 receptor alpha 2, or WT1 in combination with vaccines targeting antigens more specific to individual patients may increase the efficacy of this treatment modality. In addition, combining multiple therapeutic strategies such as a combination of vaccination and treatment with ICIs may also overcome the challenge of tumor-immune escape. Given the challenges inherent in treating GBM, a multifaceted approach will likely be necessary to ultimately generate effective immune therapies for this disease.

**Table 1:** Summary of all vaccine-based clinical trials discussed in this review.

Clinical Trial Number	Target antigen	Platform	Vehicle	Adjuvant	Reference
NCT02287428	Personalized neoantigen	Peptide		Poly-ICLC	(24)
NCT02149225	Personalized neoantigen	Peptide		Poly-ICLCGM-CSF	(23)
NCT02287428	Personalized neoantigen	Peptide		Pembrolizumab	(25)
NCT03422094	Personalized neoantigen	Peptide		Poly-ICLC Nivolumab Ipilimumab	(26)
NCT01480479	EGFRvIII	Peptide		Keyhole limpet hemocyanin GM-CSF	
NCT01498328	EGFRvIII	Peptide		Bevacizumab	(33)
NCT03299309	pp65 CMV	Peptide		Tetanus-diphtheria toxoid Montanide ISA 51	(35)
NCT04280848	TERT	Peptide		Montanide ISA 51	(38)
NCT02454634	IDH1 R132H	Peptide		Montanide Imiquimod	(41, 43)
NCT02193347	IDH1 R132H	Peptide		Tetanus-diphtheria toxoid	(42)
NCT02455557	Survivin	Peptide		Keyhole limpet hemocyanin Montanide ISA 51 GM-CSF	(45)
	WT1	Peptide		Montanide ISA 51	(48)
NCT02498665	WT1 DSP-7888	Peptide			(50)
NCT02750891	WT1 DSP-7888	Peptide			(51)
NCT03149003	WT1 DSP-7888	Peptide		Bevacizumab	(52)
NCT01130077	Survivin IL-13 receptor alpha 2 EphA2	Peptide		Poly-ICLC	(54)
NCT01222221	IMA950	Peptide		GM-CSF	(55)
NCT01920191	IMA950	Peptide		Poly-ICLC	(56)
NCT03665545	IMA950	Peptide		Poly-ICLC Pembrolizumab	(57)
NCT03491683	WT1 PSMA TERT	DNA		IL-12 Cemiplimab	(62–64)
NCT04015700	Personalized neoantigen	DNA		IL-12	(65)
NCT04573140	Tumor mRNA pp65 CMV	RNA			(68)
	EGFRvIII	Peptide	DCs	Keyhole limpet hemocyanin	(72)
	WT1 Tumor lysate	Peptide	DCs	OK-432	(73)
NCT02649582	WT1	RNA	DCs		(74)
NCT02049489	CD133	Peptide	DCs		(75)
	IL-13 receptor alpha 2	Peptide	DCs		(76)
NCT00639639	pp65 CMV	RNA	DCs	GM-CSF	(77)
NCT00639639	pp65 CMV	RNA	DCs	Tetanus-diphtheria toxoid CCL3	(78)
NCT02465268	pp65 CMV	RNA	DCs	GM-CSF Tetanus-diphtheria toxoid	(80)
NCT02529072	pp65 CMV	RNA	DCs	Nivolumab	(81)
NCT01280552	ICT-107	Peptide	DCs		(82)
NCT02546102	ICT-107	Peptide	DCs		(31)
NCT00045968	Tumor lysate	Peptide	DCs		(83)
2009-015979-27 (EudraCT)	Tumor lysate	Peptide	DCs		(84)
2006-002881-20 (EudraCT)	Tumor lysate	Peptide	DCs		(85)
NCT00323115	Tumor lysate	Peptide	DCs		(86)
NCT01006044	Tumor lysate	Peptide	DCs		(87)
2008-005035-15 (EudraCT)	Tumor lysate	Peptide	DCs		(88)
	Tumor lysate	Peptide	DCs		(89)
NCT01808820	Tumor lysate	Peptide	DCs	Imiquimod	(31)
NCT03879512	Tumor lysate	Peptide	DCs	Cyclophosphamide Nivolumab Ipilimumab	(31)
NCT04201873	Tumor lysate	Peptide	DCs	Pembrolizumab Poly-ICLC	(31)
NCT00846456	Glioma stem cells	RNA	DCs		(90)
NCT01567202	Glioma stem cells	Peptide	DCs		
NCT02010606	Glioma stem cells	Peptide	DCs		
NCT02820584	Glioma stem cells	Peptide	DCs		
NCT00293423	Tumor lysate	Peptide	HSPs		(102)
NCT03018288	Tumor lysate	Peptide	HSPs	Pembrolizumab	(103)
NCT02366728	pp65 CMV	RNA	DCs	Tetanus-diphtheria toxoid Basiliximab	(106)
NCT03927222	pp65 CMV	RNA	DCs	GM-CSF Tetanus-diphtheria toxoid	(107)
NCT01204684	Tumor lysate	Peptide	DCs	Imiquimod/Resiquimod Poly-ICLC	(108)
NCT03688178	pp65 CMV	RNA	DCs	Varlilumab Tetanus-diphtheria	(117)

## Author Contributions

All authors wrote and edited the article and approved the submitted version.

## Funding

This research was partly supported by the Intramural Research Program of the NIH, National Cancer Institute, and the Center for Cancer Research. EB is supported by a Marshall Scholarship from the Marshall Aid Commemoration Commission and the NIH-Oxford-Cambridge-Scholars Program.

## Conflict of Interest

The authors declare that the research was conducted in the absence of any commercial or financial relationships that could be construed as a potential conflict of interest.

## Abbreviations

CNS, central nervous system; GBM, glioblastoma; MDSC, myeloid-derived suppressor cell; HLA, human leukocyte antigen; IDH, isocitrate dehydrogenase; WT1, Wilms tumor 1; TERT, telomerase reverse transcriptase; MHC, major histocompatility complex; PFS, progression-free survival; OS, overall survival; EGFR, epidermal growth factor receptor; CMV pp65, cytomegalovirus phosphoprotein 65; DC, dendritic cell; GSC, glioma stem cell; HSP, heat shock protein; HSPPC, heat shock protein-peptide complex; TLR, toll-like receptor; PET, positron emission tomography; BBB, blood-brain barrier; TME, tumor microenvironment; ICI, immune checkpoint inhibitors.
